# Point mutations of the mTOR-RHEB pathway in renal cell carcinoma

**DOI:** 10.18632/oncotarget.4963

**Published:** 2015-07-20

**Authors:** Arindam P. Ghosh, Christopher B. Marshall, Tatjana Coric, Eun-hee Shim, Richard Kirkman, Mary E. Ballestas, Mitsuhiko Ikura, Mary-Ann Bjornsti, Sunil Sudarshan

**Affiliations:** ^1^ Department of Urology, University of Alabama at Birmingham, Birmingham, Alabama, USA; ^2^ Department of Pharmacology and Toxicology, University of Alabama at Birmingham, Birmingham, Alabama, USA; ^3^ Department of Pediatrics, University of Alabama at Birmingham, Birmingham, Alabama, USA; ^4^ Department of Medical Biophysics, Campbell Family Cancer Research Institute, Princess Margaret Cancer Centre, University of Toronto, Toronto, Canada

**Keywords:** mTOR, RHEB, rapamycin, renal cancer, mutations

## Abstract

Aberrations in the mTOR (mechanistic target of rapamycin) axis are frequently reported in cancer. Using publicly available tumor genome sequencing data, we identified several point mutations in *MTOR* and its upstream regulator *RHEB* (Ras homolog enriched in brain) in patients with clear cell renal cell carcinoma (ccRCC), the most common histology of kidney cancer. Interestingly, we found a prominent cluster of hyperactivating mutations in the FAT (FRAP-ATM-TTRAP) domain of mTOR in renal cell carcinoma that led to an increase in both mTORC1 and mTORC2 activities and led to an increased proliferation of cells. Several of the FAT domain mutants demonstrated a decreased binding of DEPTOR (DEP domain containing mTOR-interacting protein), while a subset of these mutations showed altered binding of the negative regulator PRAS40 (proline rich AKT substrate 40). We also identified a recurrent mutation in *RHEB* in ccRCC patients that leads to an increase in mTORC1 activity. *In vitro* characterization of this RHEB mutation revealed that this mutant showed considerable resistance to TSC2 (Tuberous Sclerosis 2) GAP (GTPase activating protein) activity, though its interaction with TSC2 remained unaltered. Mutations in the FAT domain of *MTOR* and in *RHEB* remained sensitive to rapamycin, though several of these mutations demonstrated residual mTOR kinase activity after treatment with rapamycin at clinically relevant doses. Overall, our data suggests that point mutations in the mTOR pathway may lead to downstream mTOR hyperactivation through multiple different mechanisms to confer a proliferative advantage to a tumor cell.

## INTRODUCTION

mTOR is a conserved serine/threonine kinase that integrates intracellular and extracellular signals to regulate vital cellular processes such as growth, proliferation and metabolism [1]. One of the primary characteristics of a tumor cell is its ability to disconnect growth-promoting processes from the perception of growth signals. Hence it is not surprising that elevated mTOR signaling has been detected in several cancers [2]. Recent deep sequencing efforts have identified point mutations in *MTOR* in various cancers, though it remains to be assessed if these are driver mutations causally implicated in oncogenesis [3, 4]. Understanding the regulation of the mTOR pathway is of paramount importance in renal cancer as inhibitors of mTOR (everolimus and temsirolimus) which are structural analogs of rapamycin are clinically approved for the treatment of advanced metastatic cancer.

The mTOR protein exists in two distinct multi-protein complexes: mTORC1 and mTORC2 [5]. RAPTOR (regulatory associated protein of mTOR) and RICTOR (rapamycin-insensitive companion of mTOR) are unique scaffolding proteins that assemble the complexes and bind the substrates for mTORC1 and mTORC2, respectively [6, 7]. Unique components also exist in each complex: mTORC1 comprises a negative regulator, PRAS40, whereas mTORC2 contains PROTOR (protein observed with rictor 1 and 2) and mSIN1(mammalian stress-activated map kinase-interacting protein 1) [8-10]. mTORC1 and mTORC2 share mLST8(mammalian lethal with sec-13) and the negative regulator DEPTOR [11, 12]. The complex in which mTOR participates dictates the substrate specificity of its kinase activity. S6K1 (S6 Kinase 1) and 4E-BP1 (eIF-4E binding protein 1) are two well-characterized mTORC1 substrates that associate with mRNAs and regulate both mRNA translation initiation and progression, thus enhancing protein synthesis [13, 14]. As such, mTOR is normally subject to stringent regulation by nutrient conditions [15]. The heterodimer consisting of TSC1 (tuberous sclerosis 1; also known as hamartin) and TSC2 (tuberous sclerosis 2; also known as tuberin) is a key upstream regulator of mTORC1 and functions as a GTPase-activating protein (GAP) for RHEB [16]. The GTP-bound form of RHEB directly interacts with mTORC1 and strongly stimulates its kinase activity. As a RHEB GAP, TSC1/2 negatively regulates mTORC1 by converting RHEB into its inactive GDP-bound state [17]. mTORC2 substrates include members of the AGC (protein kinase A/protein kinase G/protein kinase C) family that regulate cell survival and cell cycle progression. One of the most well characterized downstream targets of mTORC2 is AKT. mTORC2 directly activates AKT by phosphorylating its hydrophobic motif (Ser473), a site required for its maximal activation [18].

Using publicly available databases of cancer genome sequence data, we examined a cluster of mutations in *MTOR* specific to RCC located in the FAT domain of mTOR and a point mutation in the *RHEB* gene [19, 20]. These activating mutations demonstrate that multiple mechanisms may lead to mTOR hyperactivation. Our data demonstrate that mutations in the FAT domain of mTOR promote mTORC1 and mTORC2 activity. Morevoer, we demonstrate that cancer-associated *MTOR* mutations in the FAT domain confer a proliferative advantage over wild type *MTOR*. Finally, our data demonstrate that these mutations can promote persistent mTOR activity despite rapalog therapy. Therefore, these data have clinical implications given the widespread use of rapalog therapy for advanced RCC and the high prevalence of *MTOR* mutations in this malignancy.

## RESULTS

### Point mutations are clustered in various regulatory domains of mTOR in ccRCC patients and are associated with poor prognosis

**Figure 1:**
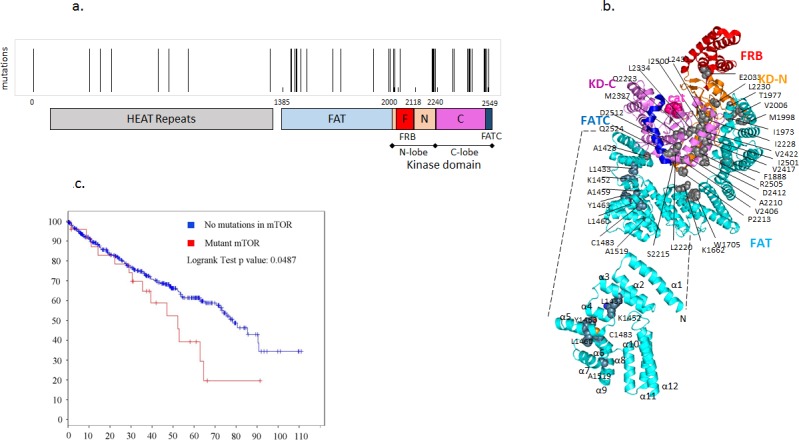
Point mutations in MTOR are clustered in various regulatory domains in ccRCCC and are associated with poor prognosis **a.** Analysis of ccRCC cases from the COSMIC and cBIO databases show that mTOR mutations are present in about 6% cases of cases. Clusters of mTOR mutations are represented in the various domains of mTOR. **b.** Sites of mTOR mutations reported in RCC are indicated as grey spheres on the structure of mTORΔN (PDB ID code: 4JSN). The catalytic site is represented with pink spheres, and the domains are colored as indicated previously. KD-N and KD-C represent the N and C terminal lobes of the kinase domain. Inset: Structure of the TRD (Tetratricopeptide repeat domains)1/2 regions (1385-1666) of the FAT domain with the sites of mutations characterized in this study represented as spheres and coloured by atom type (C, grey; O, red; N, blue; S, orange). These mutations were clustered in the kinase and FAT domains of mTOR. **c.** Survival of RCC patients as a function of *MTOR* mutation.

We analyzed cancer genomic sequence data from The Cancer Genome Atlas (TCGA) using the COSMIC (the Catalogue Of Somatic Mutations In Cancer) (http://cancer.sanger.ac.uk) and cBIO Cancer genomics portal [19-21] and found that mutations in *MTOR* were prevalent in about 6% of the patients with ccRCC while mutations in *RHEB* are relatively uncommon and present in about 1% of the patients with RCC. As with other cancers, some of these mutations clustered in key regulatory domains of mTOR such as the kinase domain and the FRB (FKBP12 rapamycin binding) domains (Table 1 and Figure 1a). Mapping the RCC-associated mutations on the three-dimensional structure of mTOR (PDB ID code: 4JSN) reveals a cluster of mutations within the core of the kinase domain, as well as several mutations distributed across the surface of the kinase domain that mediate interactions with the FAT domain (Figure 1b).

**Table 1:** Point mutations in mTOR from patients with ccRCC were annotated from the TCGA and represented by their respective domains

amino acid	mutation	domain	amino acid	mutation	domain
G5R	missense	HEAT repeats	M1998I	missense	FRB
T314N	missense	HEAT repeats	V2006L	missense	FRB
V373L	missense	HEAT repeats	E2033V	missense	FRB
G438W	missense	HEAT repeats	A2210P	missense	kinase
A695S	missense	HEAT repeats	P2213L	missense	kinase
A695D	missense	HEAT repeats	S2215Y	missense	kinase
R755H	missense	HEAT repeats	L2220F	missense	kinase
K860N	missense	HEAT repeats	Q2223L	missense	kinase
T1046T	coding-silent	HEAT repeats	I2228T	missense	kinase
L1212L	coding-silent	HEAT repeats	L2230V	missense	kinase
M1313I	missense	HEAT repeats	M2327I	missense	kinase
A1428T	missense	FAT	L2334V	missense	kinase
L1433S	missense	FAT	V2406A	missense	kinase
K1452N	missense	FAT	D2412V	missense	kinase
A1459P	missense	FAT	V2417M	missense	kinase
L1460P	missense	FAT	V2422I	missense	kinase
Y1463S	missense	FAT	L2431P	missense	kinase
Y1463Y	coding-silent	FAT	I2500M	missense	kinase
C1483F	missense	FAT	I2501F	missense	kinase
C1483Y	missense	FAT	R2505P	missense	kinase
A1519T	missense	FAT	D2512H	missense	kinase
K1662M	missense	FAT	Q2524L	missense	FATC
W1705R	missense	FAT	E2526E	coding-silent	FATC
A1835A	coding-silent	FAT			
Q1886Q	coding-silent	FAT			
F1888I	missense	FAT			
F1888L	missense	FAT			
I1973F	missense	FAT			
T1977K	missense	FAT			
T1977R	missense	FAT			

The recently elucidated crystal structure of mTOR [22] revealed that the FAT domain contains 28 α-helices arranged as α-α-helical repeats (Figure 1b). Helices α1 to α22 belong to the Tetratricopeptide (TPR) repeat family and form three discontinuous domains (TRD1, TRD2 and TRD3). Helices α23 to α28 belong to the HEAT family and form a single domain (HRD). The four domains pack sequentially to form a ‘C’-shaped α-solenoid that wraps halfway around the kinase domain and clamps onto it. mLST8 and the FRB domain protrude from the kinase domain, on opposite sides of the catalytic cleft. The FATC domain is integral to the kinase domain structure. TRD1 interacts with the C lobe on one side of the kinase domain, and after TRD2 and TRD3 traverse to the other side, the HRD interacts with both the N lobe and the C lobe of the kinase domain.

The mutations cluster in the first of three discontinuous domains (TRD1), which forms intimate interactions with the C-lobe of the kinase domain. This led us to hypothesize that mutations in the FAT domain of mTOR may lead to deregulation of mTOR kinase activity, possibly by disrupting autoinhibitory interactions and/or scaffolding sites for accessory proteins associated with the mTOR complexes. Interestingly, sequence data from 418 ccRCC patients analyzed by the cBIO portal revealed that patients with mTOR mutations exhibited a significant decrease in overall survival (Figure 1c) suggesting that mutations in *MTOR* have prognostic significance.

### FAT domain mutations lead to mTOR hyperactivation

Two previous reports have examined the role of mTOR mutations in cancer. In the first report, the authors examined six hyperactivating mutations reported in various cancers and identified a hyperactivating mutation (R2505P) in RCC [23]. In the second report, the authors examined the effects of recurrent mutations in various domains of mTOR and analyzed them with respect to their sensitivity to rapamycin [24]. Intuitively, most of the mutations reported in various cancer genome databases cluster in key regulatory domains, such as the FRB and kinase domains of mTOR. However, very little is known about the regulatory roles of other domains of mTOR and how mutations in them could alter global mTOR function and complex assembly. Based on these findings, we focused on the FAT domain cluster of *MTOR* mutants and compared its *in vitro* effects to another previously characterized mutant (R2505P) in the kinase domain [23].

**Figure 2:**
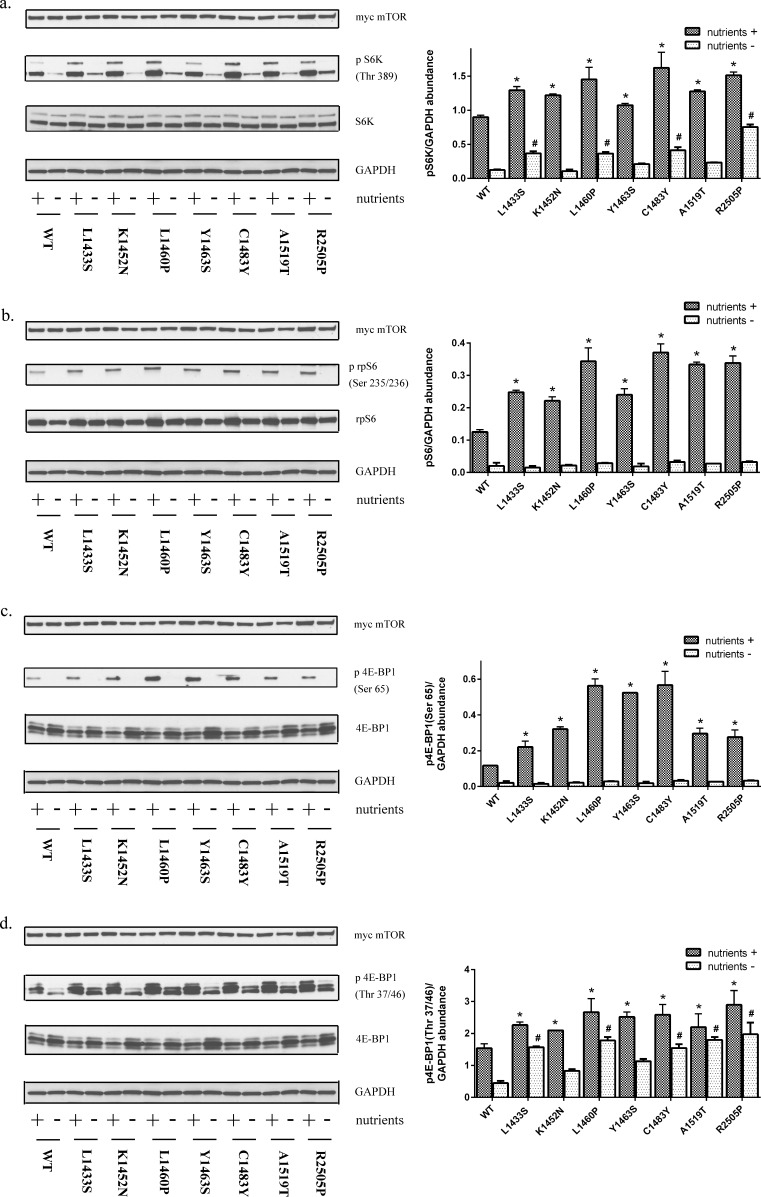
Mutations in the FAT domain promote mTORC1 activation HEK293T cell lysates expressing mutant or wild-type mTOR in the presence or absence of nutrients were immunoblotted for levels of **a.** phosphorylated S6K(Thr389) **b.** phosphorylated S6 (Ser 235/236) **c.** phosphorylated 4E-BP1 (Ser 65) and **d.** phosphorylated 4E-BP1(Thr 37/46).

To assess the sensitivity of FAT domain mutants of mTOR to upstream signaling by nutrients, we examined the effect of these mutations under nutrient replete conditions and in the absence of both serum and amino acids. We find that FAT domain mutants promote mTORC1 activation as demonstrated by the increased phosphorylation of endogenous S6K and its downstream target S6, relative to wild-type mTOR (Figure 2a and 2b, Supplemental Figures 1-3). These data are consistent with studies by Grabiner *et al*. which demonstrated that mTOR FAT domain mutants promoted phosphorylation of a cotransfected S6K1 cDNA construct [24]. Although the mTOR pathway is subject to regulation by nutrient conditions, recent studies indicate that mTORC1 phosphorylation sites have differential sensitivity to nutrients and rapamycin. The substrate quality affects how mTORC1 substrates respond to both pharmacological and natural regulators of the kinase. For example, sites such as S6K Thr389 and 4E-BP1 Ser65 are nutrient sensitive whereas 4E-BP1 Thr37/47 is relative resistant to regulation by nutrient conditions and rapamycin [25]. Our data demonstrate that mTOR FAT domain mutants promote 4E-BP1 phosphorylation at the nutrient sensitive site Ser65 in the presence of nutrients (Figure 2c, Supplemental Figures 1-3). FAT domain mutants also promote increased phosphorylation of 4E-BP1 at the nutrient resistant Thr 37/46 residue in both the presence and absence of serum and amino acids (Figure 2d, Supplemental Figure 3). Similar findings at Thr 37/46 were found in HeLa and NIH/3T3 cells under nutrient replete conditions (Supplemental Figures 1 and 2). Overall, our data demonstrate that point mutations in the FAT domain lead to activation at both rapamycin/nutrient sensitive *and* resistant outputs of mTORC1.

### Point mutations in the FAT domain of mTOR lead to increased mTORC2 *in vitro* kinase activity

**Figure 3:**
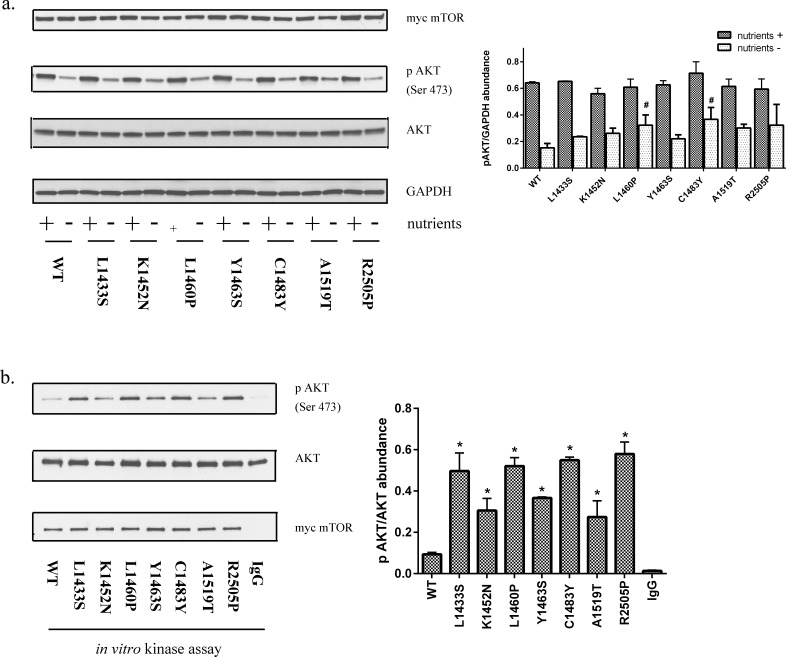
Mutations in the FAT domain promote mTORC2 kinase activity **a.** HEK293T cell lysates expressing mutant or wild-type mTOR in the presence or absence of nutrients were immunoblotted for levels of phosphorylated AKT (Ser 473). **b.** Immunoprecipitates from HEK293T cells expressing wild-type or mutant mTOR was subjected to an *in vitro* kinase assay using recombinant insect AKT as a substrate. HEK293T cells transfected with wild-type mTOR, immunoprecipitated with normal mouse IgG was used as a control.

mTORC1 and mTORC2 have been shown to play critical yet functionally distinct roles. While the functional consequences of mTORC1 activation and its downstream targets are well defined, the role of mTORC2 activation in general and its role in tumorigenesis is less established. Prior reports that have examined point mutations in mTOR, demonstrate mTORC1 activation [23, 26]. In general, we found that FAT domain mutants led to a modest increase in mTORC2 activity as determined by ser473 phosphorylation of AKT (Figure 3a). Basal AKT phosphorylation at Ser473 is high in HEK293T cells. Phosphorylation of AKT at Ser473 in the carboxy-terminal hydrophobic motif, either by mTOR [18] or by DNA-PK [27], stimulates full AKT activity. While mTORC2 is primarily responsible for this phosphorylation event, Ser473 phosphorylation is a more accurate marker of PI3K activity than mTORC2 [28]. Moreover, alternate kinases may promote phosphorylation at this site [29]. We therefore examined *de novo* AKT phosphorylation by mTORC2 by an *in vitro* kinase assay using recombinant AKT as a substrate. Our data clearly demonstrate that FAT domain mutations of mTOR lead to an increase in mTORC2 kinase activity relative to wild-type mTOR (Figure 3b). Interestingly, several of the point mutants that were previously reported to selectively phosphorylate mTORC1 substrates(L1460P, C1483F, and R2505P) clearly demonstrate increased mTORC2 kinase activity relative to wild-type mTOR (Figure 3b) [24].

### mTOR hyperactivating mutants demonstrate increased rates of cell proliferation

**Figure 4:**
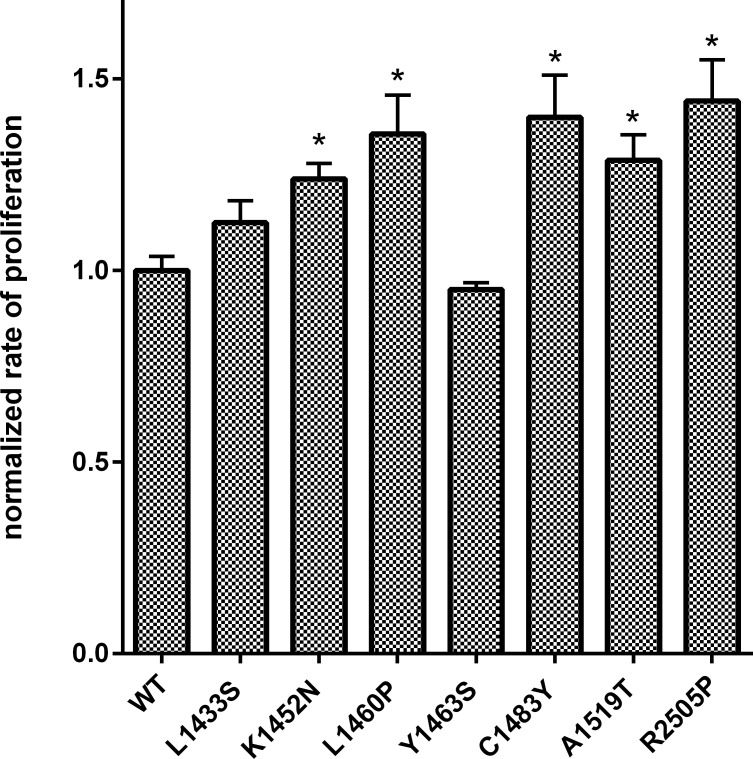
Mutations in the FAT domain of mTOR promote increased cell proliferation relative to wild-type Proliferation in cells expressing wild-type or mutant mTOR was assessed in HEK293T cells. Cells were transfected and replated at 24 hrs. Proliferation was determined 24 hours after replating. The rate of proliferation in the mutant mTOR expressing cells from biologic replicates was normalized and expressed as a fraction of the average rate of proliferation of cells expressing wild-type mTOR.

mTOR has emerged as a critical node through which cells coordinate growth signals and nutrient availability to the macromolecular synthesis of proteins, lipids and nucleic acids [30]. mTORC1 coordinates mRNA translation by phosphorylating components of the translational machinery: the eukaryotic initiation factor 4E (eIF4E)-binding proteins (4E-BPs) and the ribosomal S6 kinases (S6Ks) 1 and 2 [31]. It has been proposed that, while the 4E-BPs mediate cell proliferation downstream of mTORC1, the S6Ks regulate cell growth through complementary but distinct mechanisms [32]. As mTOR is a key regulator of cell growth and proliferation, we examined the effect of mutations in the FAT domain of mTOR on cell proliferation. *In vitro*, HEK293T cells expressing the mTOR FAT domain mutants K1452N, L1460P, C1483Y, and A1519T showed significantly higher rates of cell proliferation in comparison to cells expressing wild-type mTOR, similar to the kinase domain mutant R2505P (Figure 4). Our data demonstrates for the first time that mTOR FAT domain mutants confer a proliferative advantage relative to wild-type mTOR.

### mTORC1/2 hyperactivating mutants demonstrate differential complex assembly

**Figure 5:**
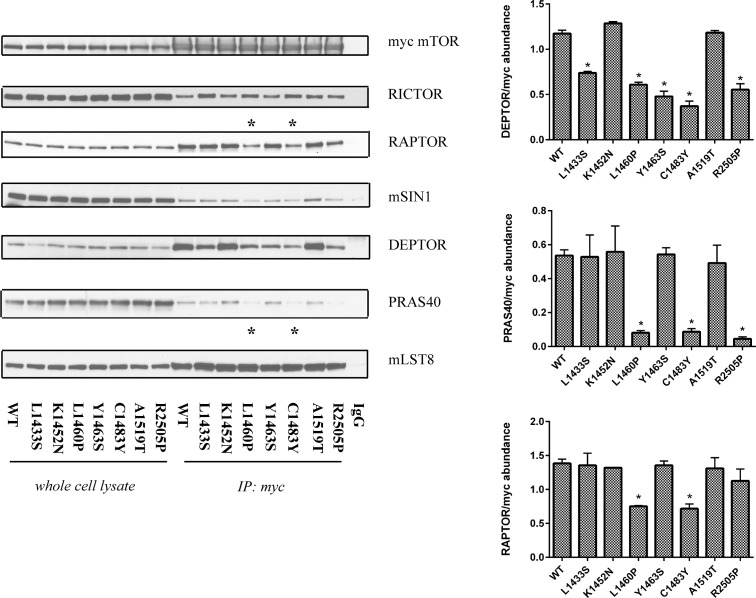
Mutations in the FAT domain of mTOR lead to altered DEPTOR and PRAS40 binding Wild-type and mutant mTOR complexes were immunoprecipitated using a myc antibody, washed and analyzed for relative levels of mTOR complex proteins. HEK293T cells transfected with wild-type mTOR, immunoprecipitated with normal mouse IgG was used as a control. The input controls comprised 10% of the lysates used for IP.

One potential mechanism by which mutations in mTOR might impact kinase activity is by altering mTORC1 and mTORC2 complex assembly. To test this hypothesis, we transiently overexpressed wild-type and mutant mTOR in HEK293T cells and immunoprecipitated the mTOR complex using the myc epitope. In contrast to the previous findings, we observed that a few of the FAT domain mutants (L1460P and C1483Y) had lower levels of RAPTOR in the immunoprecipitates in comparison to wild-type mTOR (Figure 5) [24]. By binding to RAPTOR, PRAS40 is known to regulate mTORC1 kinase activity by functioning as a direct inhibitor of substrate binding [33]. We examined the immunoprecipitates from wild-type and mutant mTOR for levels of PRAS40. Notably, the same mutants demonstrated a loss of PRAS40 binding. Our data thus suggests that some of the mutations in the FAT domain of mTOR may preferentially lead to an increased phosphorylation of mTORC1 substrates due to loss of PRAS40 binding.

In addition, we also observed an alteration in the levels of RICTOR in the immunoprecipitates from mutant mTOR in comparison to its wild-type counterpart (Figure 5). RICTOR is a conserved scaffolding protein that regulates the recruitment of substrates to the mTORC2 complex [18, 34]. Our mTORC2 complex immunoprecipitation data correlates with our results from the *in vitro* kinase assay in that mutants with higher *in vitro* kinase activity (L1433S, L1460P, and C1483Y) have high RICTOR levels in immunproprecipitates. Notably, the R2505P mutant did not demonstrate an increase in RICTOR binding. These data indicate that mutations in the FAT domain of mTOR may lead to an overall increase in mTORC2 kinase activity due to better recruitment of mTORC2 substrates via enhanced RICTOR association. As previously reported, we observed a loss of DEPTOR binding in several of the FAT domain mutants relative to wild-type mTOR (Figure 5) [24]. As DEPTOR is an intrinsic inhibitor common to mTORC1 and mTORC2 complexes, these findings further substantiate our results from the mTORC2 *in vitro* kinase assay. Overall, the immunoprecipitation experiments from cells over expressing wild-type and mutant mTOR suggest that point mutations in mTOR may act through several mechanisms to increase the phosphorylation of downstream mTORC1 and mTORC2 targets.

### FAT domain mutations in mTOR confer relative resistance to mTOR pathway inhibitors

**Figure 6:**
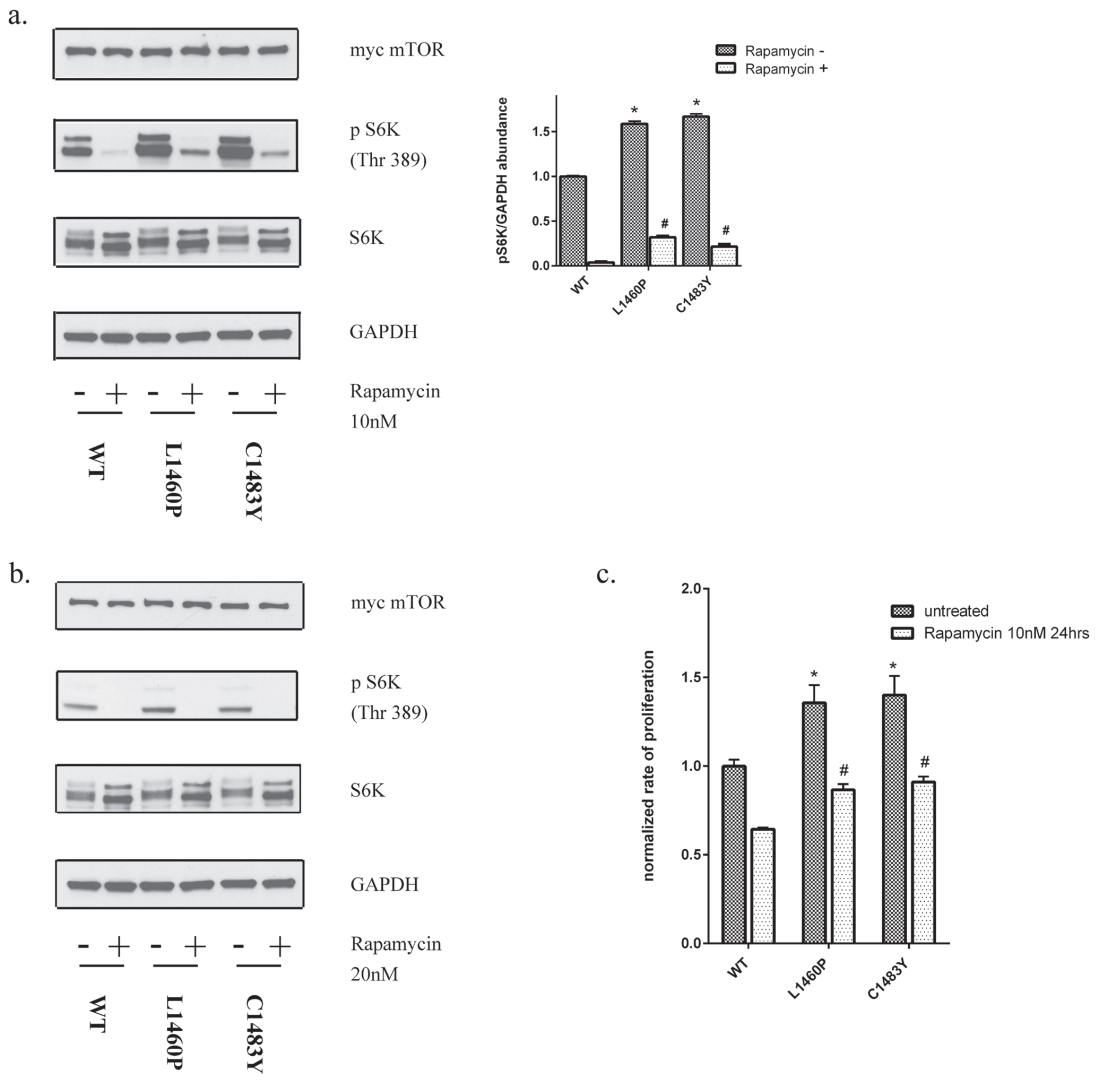
Mutations in the FAT domain of mTOR lead to decreased sensitivity to the inhibitory effects of rapamycin at clinically relevant doses **a.** HEK293T cells overexpressing wild-type or mutant mTOR were treated with 10nM rapamycin for 30 mins and protein lysates were immunoblotted for the indicated proteins. **b.** HEK293T cells overexpressing wild-type or mutant mTOR were treated with 20nM rapamycin for 60 mins and protein lysates were immunoblotted for the indicated proteins. **c.** Cell proliferation was assessed as described previously in cells expressing wild-type or mutant mTOR in the presence of rapamycin (10nM for 24hrs).

Recent studies have indicated that mutations of the mTOR pathway may have therapeutic implications. This is particularly relevant as rapalogs are currently used for the treatment of advanced RCC [35]. Prior studies in cell lines have implicated mTOR mutations as conferring sensitivity to rapamycin [24]. However, a recent case report implicated mTOR mutation (F2108L) as a mechanism of acquired resistance to allosteric inhibition by the rapalog everolimus [36]. These data prompted us to examine the effects of rapamycin on mTORC1 activity in cells expressing the hyperactivating FAT domain mutants. To assess the sensitivity of mTOR mutants to rapalogs, we treated the cells with rapamycin at 10nM which is comparable to trough levels of patients with advanced RCC treated with everolimus [37]. While the mTORC1 activity of FAT domain mutants was inhibitable by rapamycin, multiple mutants demonstrated residual activity as determined by S6K phosphorylation (Figure 6a). With higher doses of rapamycin, this residual activity could be abolished (Figure 6b). To assess the sensitivity of these mutants to rapamycin we also measured proliferation of these cells expressing mutant mTOR relative to wild-type over expressing cells in the presence of rapamycin. Notably, HEK293T cells expressing FAT domain mutants (L1460P and C1483Y) demonstrated higher rates of cell proliferation in comparison to wild-type mTOR expressing cells in the presence of clinically relevant doses of rapamycin (Figure 6c).

### A point mutation in RHEB leads to mTOR activation in a subset of patients with RCC

In the abundance of growth factors activation of the PI3K(phosphatidylinositol 3-kinase) activates and phosphorylates AKT, that in turn leads to the downstream phosphorylation of TSC2 and inhibits its GAP activity towards RHEB, allowing RHEB to activate mTOR [38, 39]. Amino acids on the other hand signal to mTORC1 through the Rag family of GTPases, by mediating the activation of mTOR by RHEB at the lysosome [12, 40]. Our analysis of cancer genome databases, also led us to identify a recurrent point mutation in RHEB (Y35N), reported in RCC and in several other cancers [41, 42]. RHEB is responsive to growth factors, but, in conjunction with PLD1 (phospholipase D1), is also an integral part of the machinery that stimulates mTORC1 in response to amino acids [43]. In the simplest model, GTP-bound RHEB either stimulates the kinase activity of mTORC1 via direct interaction or induces a conformational change in mTORC1 that results in enhanced substrate turnover [44]. RHEB has an unusually slow intrinsic GTPase activity, which is regulated by the GAP activity of TSC2 [45]. Previous data utilizing site-directed mutagenesis, crystallography, and real-time NMR-based GTPase assays suggest that RHEB Tyr35 is a structurally critical residue that is highly conserved across the small GTPase superfamily [46], but has unique functions in RHEB. Tyr35 was found to maintain the high activation state of RHEB by inhibiting intrinsic GTP hydrolysis, however it was also found to be required for sensitivity to the GAP activity of TSC2 [47].

**Figure 7:**
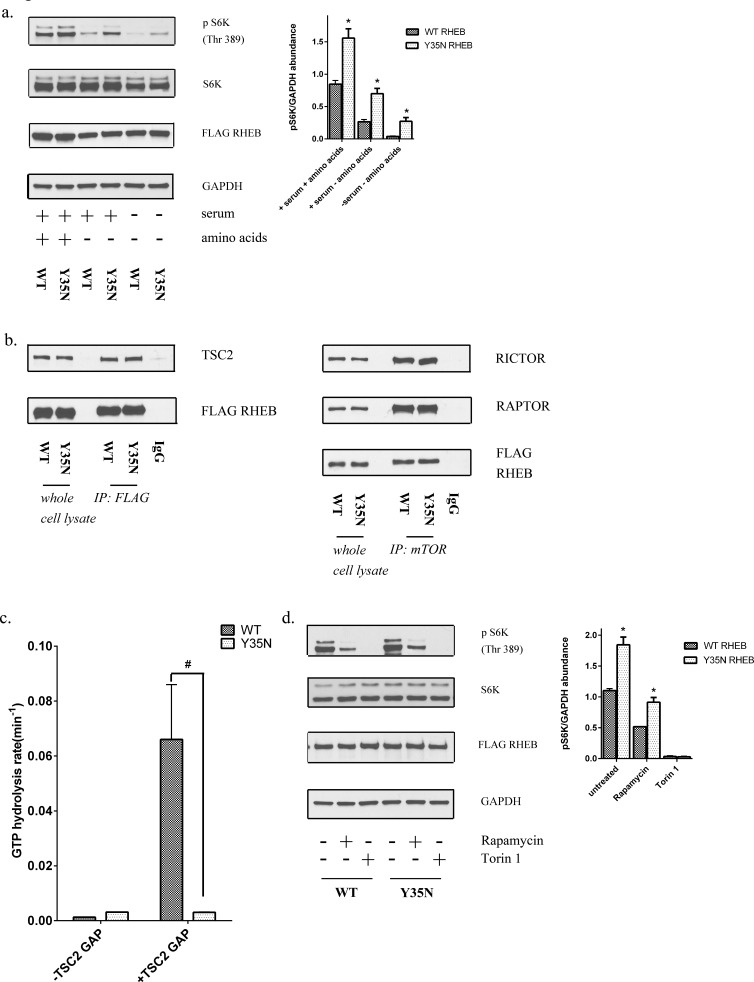
Y35N RHEB mutation in ccRCC causes mTORC1 hyperactivation by resistance to TSC2 GAP activity **a.** HEK293T cells expressing FLAG-tagged wild-type or mutant RHEB were analyzed for levels of phosphorylated mTORC1 downstream targets in the presence and absence of nutrients. **b.** Immunoprecipitates from wild-type or mutant RHEB overexpressing cells were analyzed for relative levels of RICTOR, RAPTOR and TSC2. HEK293T cells transfected with wild-type RHEB, immunoprecipitated with normal mouse IgG was used as a control **c.** Hydrolysis of GTP in the presence of absence of TSC2 GAP domain in wild-type and Y35N RHEB. Results from three independent experiments were normalized to the rate of GTP hydrolysis of wild-type RHEB. # represents a *p* value of ≤ 0.05 in the GTP hydrolysis rate of Y35N RHEB in comparison to wild-type RHEB in the presence of TSC2 GAP domain. **d.** Cells expressing FLAG-tagged wild-type or mutant RHEB were treated with rapamycin (10nM) for 30 mins and protein lysates were immunoblotted for the indicated proteins.

To assess the role of a point mutation in RHEB at the conserved Tyr35 site, we generated a point mutation (Y35N) and transiently over expressed wild-type and mutant RHEB in HEK293T cells. As RHEB is a key mediator of the amino acid sensing mechanism of the cell, we examined the effect of this mutant in the presence and absence of amino acids. Mutant RHEB caused promoted mTORC1 activation under nutrient containing conditions, an effect that remained under nutrient deprived conditions as demonstrated by persistent phosphorylation of S6K and 4E-BP1 (Figure 7a). RHEB stimulates mTORC1 phosphorylation of S6K and 4E-BP1 in a rapamycin-sensitive manner [48]. To determine the mechanism by which a point mutation in RHEB might lead to downstream mTORC1 activation, we first examined whether mutant RHEB altered assembly of mTOR complexes by immunoprecipitating endogenous mTOR complexes using an anti-mTOR antibody from cells overexpressing wild-type or mutant RHEB. As expected, overexpression of mutant RHEB did not alter the association of RAPTOR or RICTOR, indicating the mutation does not impact mTORC1 or mTORC2 assembly, respectively (Figure 7b).

The TSC1/TSC2 complex interacts with RHEB and inactivates it through the activity of a GAP domain in TSC2. Thus we performed immunoprecipitation experiments to analyze the interactions of wild-type and mutant FLAG-tagged RHEB with TSC2. The RHEB mutation did not affect interaction with TSC2 (Figure 7b). RHEB has very low intrinsic GTPase activity and our previous results have shown that the Y35 residue autoinhibits intrinsic GTP hydrolysis [47]. Using a real-time NMR-based GTPase assay, we previously found that mutation of Y35 to alanine or phenylalanine disrupted this autoinhibitory mechanism and accelerated intrinsic GTP hydrolysis several fold, while simultaneously rendering it insensitive to the GAP activity of TSC2 [47]. Similar to the engineered Y35A and Y35F mutations, we found that the cancer-associated Y35N RHEB mutation increased the rate of intrinsic GTP hydrolysis by ~2.5 fold, due to release of the autoinhibitory function of Y35. Subsequently we tested the sensitivity of the GTPase activity of Y35N RHEB to the action of the TSC2 GAP domain. While the rate of GTP hydrolysis of wild-type RHEB was increased 50-fold in the presence of the GAP domain of TSC2, the Y35N mutant exhibited complete resistance to the GAP domain of TSC2 with no detectable effect on GTP hydrolysis (Figure 7c). As expected, the RHEB mutant demonstrated somewhat higher levels of intrinsic GTP hydrolysis, however acquired resistance to the GAP activity of TSC2 has a more profound impact of RHEB function, thus leading to hyperactivation of mTORC1 under starvation conditions where TSC2 is active.

RHEB activation of S6K and 4E-BP1 is known to be rapamycin-sensitive [48]. To determine the rapamycin sensitivity associated with this RHEB mutant, we transiently overexpressed wild-type and mutant RHEB and examined the phosphorylation of mTOR targets after acute rapamycin treatment. While mTORC1 activity in cells expressing the RHEB mutant remained sensitive to rapamycin, higher levels of phosphorylated S6K after rapamycin treatment were observed with the mutant than wild-type RHEB (Figure 7d).

## DISCUSSION

mTOR deregulation is observed in multiple sporadic cancer types; however, it also plays a causative role in various familial tumor syndromes such as Cowden syndrome, Peutz-Jeghers syndrome and Tuberous Sclerosis as recently summarized [49]. In sporadic cancers, mTOR activation is frequently the result of amplification/activating mutations in genes encoding upstream RTK's (receptor tyrosine kinases [50], activating mutations of PI3KCA (i.e., the gene encoding the PI3K catalytic subunit p110α) [51] or deletion/inactivation of tumor suppressors, including PTEN (phosphatase and tensin homolog) [52], LKB1 (liver kinase B1) [53], and the protein phosphatase PP2A, which dephosphorylates and inactivates AKT [54].

Recent cancer genome sequencing efforts have led to the identification of point mutations in mTOR. In this study, we chose to characterize a group of mutations in the TRD1 and TRD2 regions of the FAT domain that cluster distal from the kinase domain. While mutations in these regions could directly disrupt the binding sites for mTOR complex proteins that interact with the FAT domain, these mutation sites are not surface exposed. The FAT domain is comprised of helices arranged in α-α-helical repeats that form a C-shaped solenoid that surrounds the kinase domain. These mutation sites are found at the interfaces between adjacent helices, thus we propose these RCC-associated mutations are likely to destabilize the structure of the FAT domain. This may directly deregulate mTOR kinase activity as well as affect the binding of mTOR complex proteins. The binding of DEPTOR, which interacts with the FAT domain, was impaired by four of the six mutations characterized in this study. The side chain of L1433 in α3 is involved in hydrophobic interactions with the alkyl region of K1452 in α4, which appears to stabilize the orientation of helices 3 and 4, and would be disrupted by the L1433S or K1452N mutations. Likewise, the side chains of Y1463 in α5 and C1483 in α6 are intimately involved in the α5-α6 interhelical packing, which could be disrupted by loss of an aromatic ring (Y1463S) or steric clashes (C1483F). L1460 is in the middle of α5, thus its mutation to the helix-breaker residue proline would be expected to destabilize these helical bundles, as would the adjacent mutation A1459P. A1519, located in α8, packs into a small cavity formed by α7 that would not accommodate a larger sidechain. Thus the α7-α8 this bundle would likely be disrupted by the A1519T mutation. Mechanistically, we find that in addition to altering mTORC1 and mTORC2 complex assembly, FAT domain mutations also lead to a decrease in PRAS40 and DEPTOR binding, suggesting that multiple mechanisms may act in concert to activate downstream mTOR targets.

Additionally, we examined a recurrent point mutation in *RHEB* that caused mTORC1 hyperactivation by conferring resistance to the GAP activity of TSC2. Mutations in RHEB are uncommon in many cancers, despite its central role in regulating downstream mTOR activation. In RCC, a recurrent mutation in RHEB at the Tyr35 residue has been reported, though it is unclear if this mutation is mutually exclusive to mutations in *TSC1* or *TSC2* as the mutation frequencies amongst these genes is low. The recurrent mutation of RHEB Y35 in cancers underscores the importance of this residue, and illustrates that the unique biochemical properties of RHEB lead to a different mutational hotspot relative to RAS, in which the majority of mutations affect G12, G13 and Q61. Indeed, the equivalent positions in RHEB are either already substituted in the wild-type sequence (R15 and S16), or lack a catalytic function (Q64). It is notable that while oncogenic RAS mutations synergistically impair both intrinsic hydrolysis and GAP sensitivity, the RHEB Y35N mutation enhances intrinsic hydrolysis but abolishes sensitivity to the GAP. Overall, our results suggest that mutations in the TSC-RHEB-mTOR signaling axis may lead to a loss of inhibitory inputs thus conferring a survival advantage to a dividing tumor cell.

Our findings have several implications for patients with RCC. The finding that *MTOR* mutations are associated with worsened outcomes has prognostic relevance and suggests that this event may be a significant contributor to tumor progression. Consistent with this possibility is our data demonstrating the first evidence that cancer-associated *MTOR* mutations promote a proliferative advantage over wild type *MTOR*. The TCGA data set are from primary RCC tumor samples. In the setting of RCC, the morbidity and mortality is primarily associated with disease outside the kidney. Hence, the incidence of *MTOR* mutations may be much higher in metastatic tumor sites which would further support a role for this genetic alteration in tumor progression. Additionally, our data may have therapeutic implications. Approved mTOR inhibitors temsirolimus and everolimus serve as important therapeutic options within the current RCC treatment paradigm, though they primarily target the mTORC1 pathway. Biomarkers for rapamycin sensitivity/resistance are lacking. Our results suggest that FAT domain mutations may potentially lead to resistance to rapalog therapy in patients with ccRCC. Notably, a recent case report in anaplastic thyroid cancer suggests that *MTOR* mutation is a mechanism of acquired resistance to rapalog therapy [55]. Sequencing of the pretreatment tumor demonstrated a nonsense *TSC2* mutation. After significant response to everolimus, the patient developed resistant tumor that had a *MTOR* kinase domain mutation (F2108L). Such resistance may occur in the setting of FAT domain mutations and could occur via several mechanisms as supported by our data. First, higher doses of rapamycin may be required to ablate rapamycin sensitive outputs of mTORC1. Prior *in vitro* studies have examined the rapamycin sensitivity of mTORC1 activation mediated by mTOR point mutants. However, in many cases, the doses used are much higher than serum rapalog levels in patients undergoing treatment in the setting of advanced RCC. The recent case report in thyroid cancer demonstrated persistent mTORC1 activity of the F2108L mutant at physiologically relevant rapamycin concentrations is consistent with our studies of FAT domain mutants. Additionally, it is now well established that there are mTORC1 activities that are not inhibitable by rapamycin. Our data demonstrate enhanced phosphorylation of such (e.g. 4E-BP1 Thr 37/46). Finally, mTOR FAT domain mutants can promote mTORC2 kinase activity-a critical finding given that rapalogs generally do not ablate mTORC2 activity [56]. Hence, our findings suggest that alternative strategies may need to be considered in the setting of *MTOR* mutation. Dosing adjustments could be considered. Alternatively, strategies that target rapamycin resistant activities of mTOR mediated by mTORC1 or mTORC2 (e.g ATP competitive inhibitors) could be considered.

In summary, our findings shed new insight into the biology of mTOR as it pertains to kidney cancer. While mTOR is one of the largest genes in the human genome, the mutations in mTOR are clustered within finite regulatory domains suggesting that these mutations lead to a gain of function which is currently an area of active investigation. Characterizing these mutations and their sensitivity to targeted agents could thus be clinically relevant, especially with the increasing use of whole genome sequencing to direct therapy in patients with advanced cancer.

## MATERIALS AND METHODS

### Cell lines transfections and cell treatments

HEK293T cells were obtained from American Type Culture Collection (ATCC) and maintained in DMEM supplemented with 10% heat-inactivated fetal bovine serum at 37°C in a humidified 5% CO2 atmosphere. HEK293T cells were transfected with myc-epitope tagged wild-type mTOR or mutant mTOR cDNA in their expression vectors, followed by whole cell lysis 48 hrs after transfection. For mutant RHEB experiments, HEK293T cells were transfected with FLAG-epitope tagged wild-type RHEB or mutant RHEB cDNA in their expression vectors, followed by whole cell lysis 48 hrs after transfection. Cells were starved of nutrients by gently rinsing the cells in serum free DMEM once and incubating them in serum free DMEM overnight. For amino acid starvation, cells were incubated in amino acid-free RPMI for 50 minutes as previously described [40].

### Reagents and chemicals

Rat wild-type myc mTOR cDNA was obtained from Dr. David Sabatini (Whitehead Institute - MIT) via Addgene(#1861). Human wild-type Flag RHEB cDNA was obtained from Dr. Fuyuhiko Tamanoi (UCLA) via Addgene (#19996). Protein G sepharose beads were obtained from GE Healthcare Lifesciences. Rapamycin was purchased from Selleckchem. Inactive, N-terminal His-tagged recombinant full-length human Akt1 for use in kinase assays was obtained from EMD Millipore (#14-279).

### Site-directed mutagenesis

All mTOR and RHEB point mutations were generated in the parental vectors using site-directed mutagenesis with the QuikChange II XL kit according to the manufacturer's protocol. Point mutations were verified using Sanger sequencing.

### *In vitro* proliferation assays

Six replicates each of 3000 cells were seeded into 96-well plates and assayed using Cell Titer Glo according to the manufacturer's protocol.

### Cell lysis, immunoprecipitation and kinase assays

Cells were rinsed once with ice-cold PBS and lysed in ice-cold lysis buffer (40 mM HEPES [pH 7.4], 2 mM EDTA, 10 mM pyrophosphate, 10 mM glycerophosphate, and 0.3% CHAPS or 1% Triton X-100, supplemented with protease and phosphatase inhibitors. The soluble fractions of cell lysates were isolated by centrifugation at 13,000 rpm for 10 minutes by centrifugation in a microfuge. For immunoprecipitations of myc mTOR complexes, the lysates were prepared in lysis buffer containing 0.3% CHAPS to preserve the integrity of the mTOR complexes [57]. Lysates were incubated with an antibody to myc (clone 9E10-Sigma) with rotation for 1.5 hours at 4°C. For immunopreciptation of native mTOR complexes, lysates prepared in 0.3% CHAPS lysis buffer were similarly incubated with 20 μl of goat-anti-mTOR antibody (N-19, Santacruz). 40 μl of 50% slurry of protein G-sepharose was then added and the incubation continued for an additional 1 hour. Immunoprecipitates were washed three times with ice-cold CHAPS lysis buffer, denatured by the addition of 40 μl of sample buffer and boiling for 5 minutes, resolved by SDS-PAGE, and analyzed by immunoblotting. For immunoprecipitation of FLAG tagged proteins lysates were prepared in lysis buffer containing 1% Triton-X. Lysates with incubated with 30 μl of 50% slurry of Anti-FLAG (M2) affinity gel (Sigma) with rotation for 2 hours at 4°C. Finally, the beads were washed 3 times with ice-cold lysis buffer and FLAG-tagged proteins were eluted by incubating the beads with 40 μl of sample buffer and boiling for 5 minutes, resolved by SDS-PAGE, and analyzed by immunoblotting.

The mTORC2 kinase *in vitro* kinase assay was performed as previously described [58] Briefly, immunoprecipitates from myc tagged wild-type and mutant mTOR, captured by protein G-agarose were washed four times with the CHAPS-containing lysis buffer and once with the rictor-mTOR kinase buffer (25 mm HEPES, pH 7.5, 100 mm potassium acetate, 2 mm MgCl2). For kinase reaction, immunoprecipitates were incubated in a final volume of 15 μl at 37 °C for 20 min in the rictor-mTOR kinase buffer containing 500 ng of inactive AKT1-GST and 500 μM ATP. The reaction was stopped by the addition of 235 μl of ice-cold dilution buffer (20 mm MOPS, pH 7.0, 1 mm EDTA, 0.3% CHAPS, 5% glycerol, 0.1% 2-mercaptoethanol, 1 mg/ml BSA). After a quick spin, the supernatant was removed from the protein G-agarose, and a 15-μl portion was analyzed by immunoblotting for Ser(P)473-Akt and total Akt detection. The pelleted protein G-agarose beads were also analyzed by immunoblotting to determine the levels of myc in the immunoprecipitates.

### Immunoblotting

All immunoblot analyses were performed as previously described [59]. Antibodies were obtained from the following sources: antibodies to phospho-T389-S6K1, phospho-S473-AKT, phospho-S235/236-S6, phospho-S65-4E-BP1, 4E-BP1 S6K1, AKT, S6, GAPDH, RICTOR, RAPTOR, DEPTOR, PRAS40, mLST8, mSIN1 were obtained from Cell Signaling Technology. Antibodies to myc (9E10) and FLAG (M2) were obtained from Sigma.

### Statistical analysis

Densitometric analysis of immunoblots from minimum three independent experiments normalized to loading control are shown. Error bars represent the SEM (standard error of mean). * represents a *p* value ≤ 0.05 in comparison to wild-type mTOR or RHEB overexpressing cells. # represents a *p* value ≤ 0.05 in comparison to wild-type mTOR or RHEB overexpressing cells cells starved of nutrients or treated with rapamycin.

### NMR-Based GTPase assays

Constructs encoding wild-type and Y35N human RHEB (residues 1-169) were subcloned into PGEX2T to generate thrombin-cleavable GST fusion proteins. As described previously [60], ^15^N RHEB was expressed in in E. coli BL21 DE3 Codon+ grown in M9 minimal media supplemented with ^15^N ammonium chloride, and purified on glutathione-Sepharose, released from the resin by thrombin cleavage followed by gel filtration chromatography on Sephadex-75 resin (GE Healthcare). To assay GTP hydrolysis, ^15^N-Rheb was loaded with GTP in the presence of EDTA, and excess nucleotide and EDTA were removed by passing the sample through a desalting column (PD Midi-Trap G25, GE Healthcare) (as previously described [47], and aliquots of 0.2 mM ^15^N RHEB-GTP (residues 1-169) in NMR buffer (20 mM HEPES, 100 mM NaCl, 5 mM MgCl_2_, 2 mM DTT, 10% D_2_O, pH7) were snap frozen and stored at −80°C. The assay was initiated by thawing a 35 μl sample, and successive ^15^N ^1^H HSQC spectra (8 scans, 20 min each) were collected at 15°C on a 600 MHz Bruker Avance III NMR spectrometer equipped with a 1.7 mm microcryoprobe. Pairs of GTP/GDP-specific peaks from several residues were used to evaluate the fraction of GTP-bound RHEB remaining at each time point to obtain the half-life and exchange rate as described previously [61]. To assay the GAP activity of TSC2 on wild-type and Y35N RHEB, the GAP domain (residues 1525-1742) was prepared as described previously [60] and added at a 1:2 molar ratio to a ^15^N RHEB-GTP sample prior to collection of ^15^N ^1^H HSQC spectra.

## SUPPLEMENTARY MATERIAL FIGURES


