# Supplementary material for: Point mutations of the mTOR-RHEB pathway in renal cell carcinoma
Source: Oncotarget. 2015 Jul 20;6(20):17895–910. doi: 10.18632/oncotarget.4963 (PMC4627224; doi:10.18632/oncotarget.4963)
Supplement: Supplementary file 1 [file oncotarget-06-17895-s001.pdf]

Point mutations of the mTOR-RHEB pathway in renal cell carcinoma

Supplementary Material

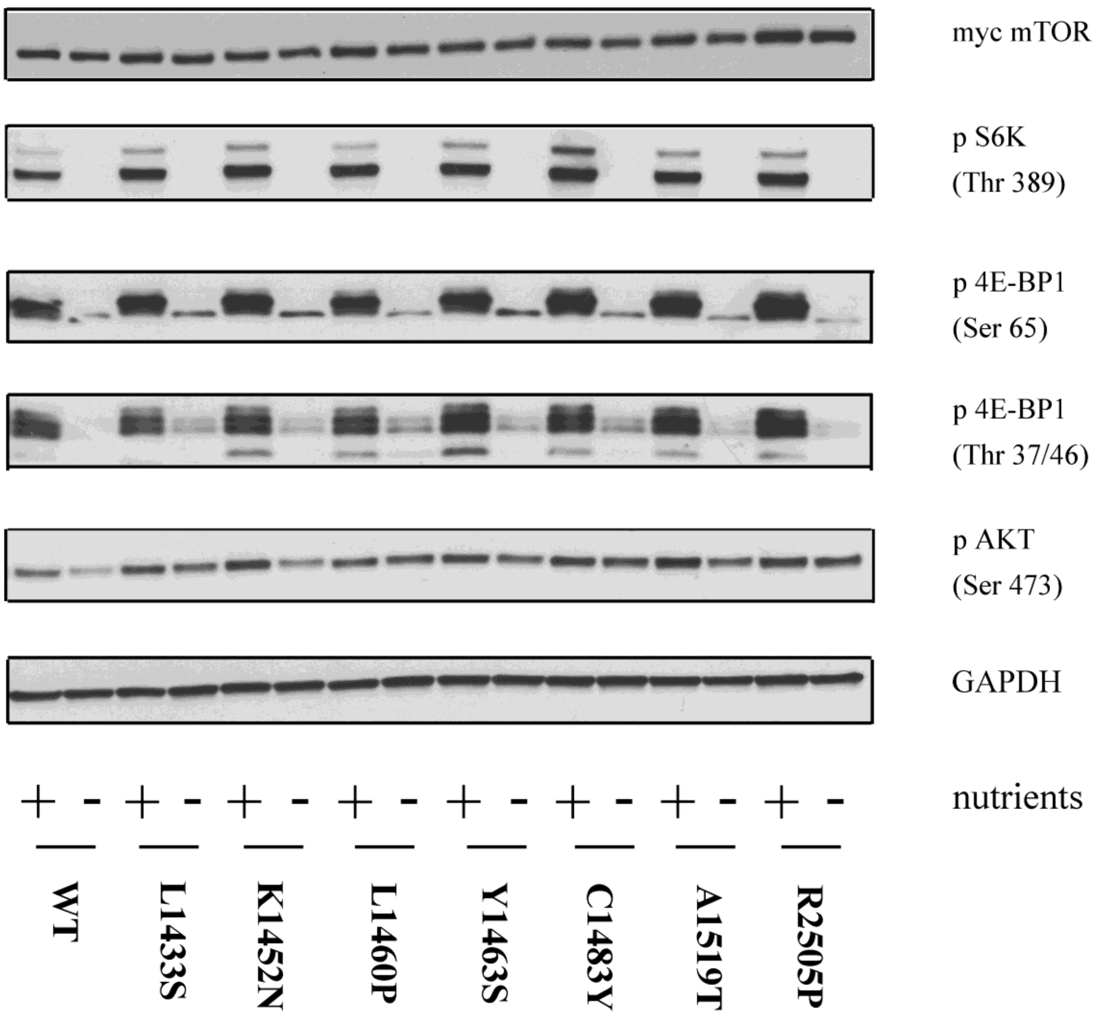

Supplemental Figure 1. Mutations in the FAT domain primarily promote mTORC1 activation in Hela cells.

HeLa cell lysates expressing mutant or wild-type mTOR in the presence or absence of nutrients were immunoblotted for levels of phosphorylated S6K(Thr389), phosphorylated 4E-BP1 (Ser 65), phosphorylated 4E-BP1(Thr 37/46) and phosphorylated AKT (Ser 473).

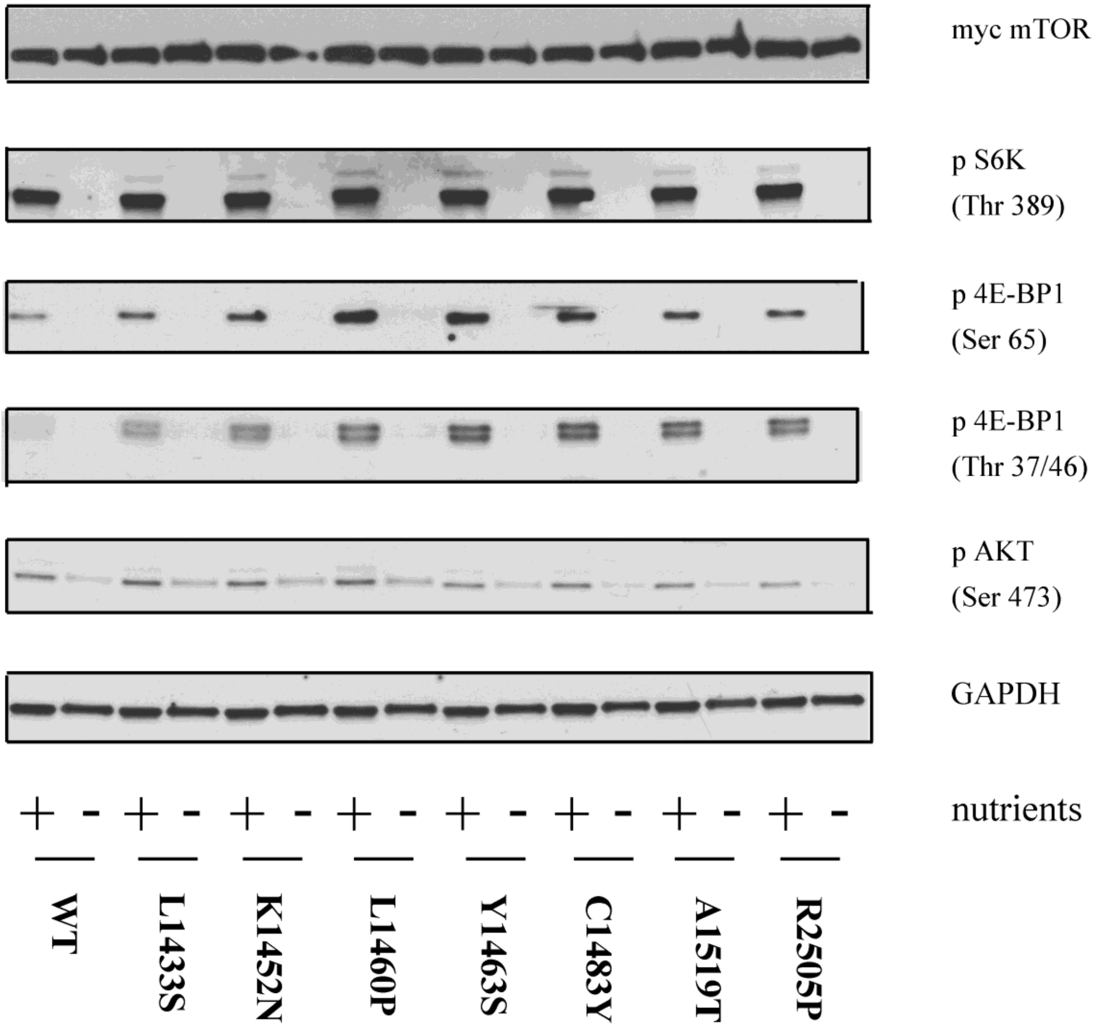

Supplemental Figure 2. Mutations in the FAT domain primarily promote mTORC1 activation in NIH/3T3.

NIH/3T3 cell lysates expressing mutant or wild-type mTOR in the presence or absence of nutrients were immunoblotted for levels of phosphorylated S6K(Thr389), phosphorylated 4E-BP1 (Ser 65), phosphorylated 4E-BP1(Thr 37/46) and phosphorylated AKT (Ser 473)

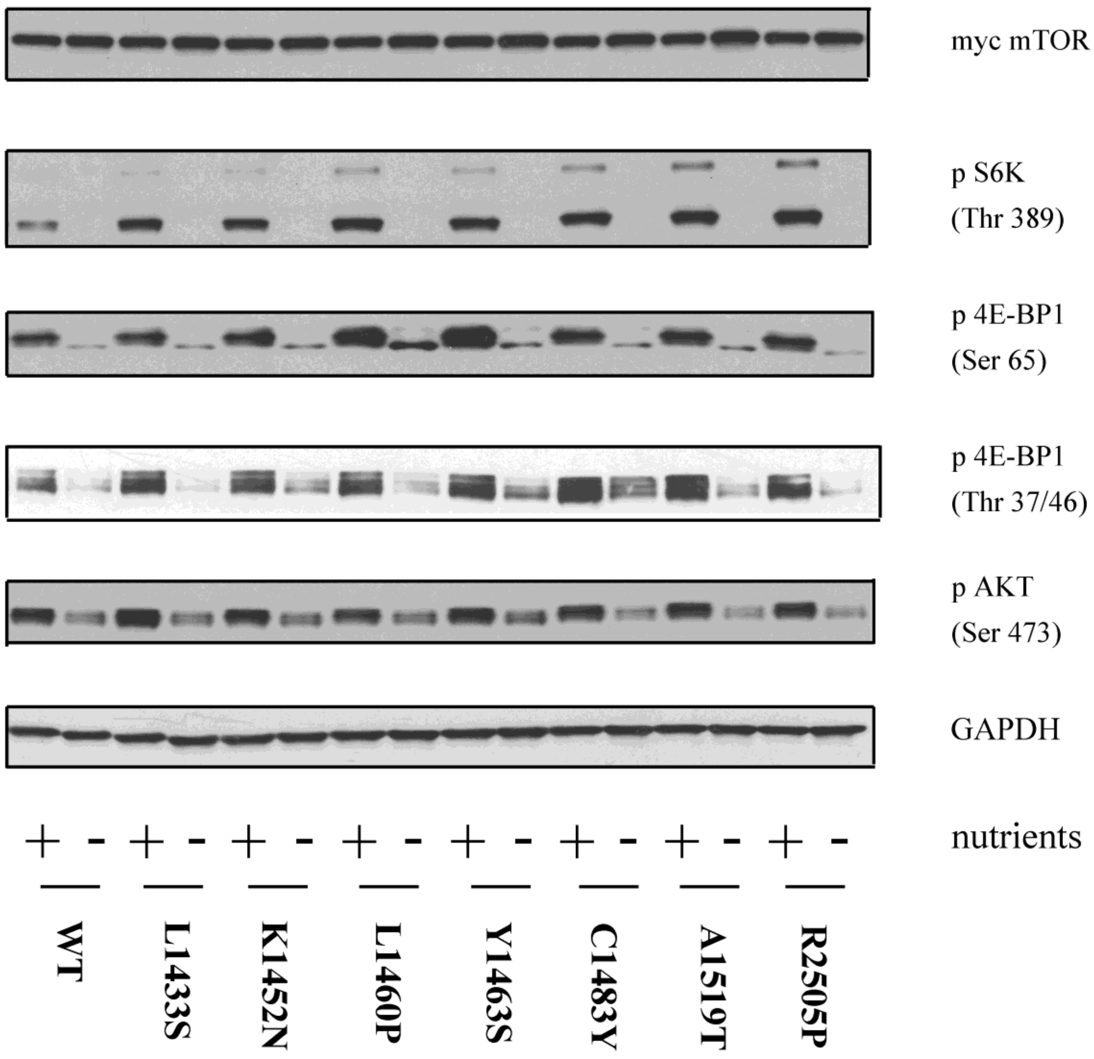

Supplemental Figure 3. Mutations in the FAT domain primarily promote mTORC1 activation in HEK293T cells.

Replicate data from HEK293T cell lysates expressing mutant or wild-type mTOR in the presence or absence of nutrients were immunoblotted for levels of phosphorylated S6K(Thr389), phosphorylated 4E-BP1 (Ser 65), phosphorylated 4E-BP1(Thr 37/46) and phosphorylated AKT (Ser 473)
